# Results of an interlaboratory method performance study for the size determination and quantification of silver nanoparticles in chicken meat by single-particle inductively coupled plasma mass spectrometry (sp-ICP-MS)

**DOI:** 10.1007/s00216-017-0427-2

**Published:** 2017-06-20

**Authors:** Stefan Weigel, Ruud Peters, Katrin Loeschner, Ringo Grombe, Thomas P. J. Linsinger

**Affiliations:** 1 RIKILT – Wageningen UR, Akkermaalsbos 2, 6708 WB Wageningen, The Netherlands; 2 Federal Institute for Risk Assessment (BfR – Bundesinstitut für Risikobewertung), Max-Dohrn-Str. 8-10, 10589 Berlin, Germany; 3 National Food Institute, Technical University of Denmark, Mørkhøj Bygade 19, 2860 Søbor, Denmark; 4 European Commission, Joint Research Centre, Directorate F – Health, Consumers and Reference Materials, Retieseweg 111, 2440 Geel, Belgium

**Keywords:** Silver nanoparticle, Single-particle ICP-MS, Food, Method validation

## Abstract

Single-particle inductively coupled plasma mass spectrometry (sp-ICP-MS) promises fast and selective determination of nanoparticle size and number concentrations. While several studies on practical applications have been published, data on formal, especially interlaboratory validation of sp-ICP-MS, is sparse. An international interlaboratory study was organized to determine repeatability and reproducibility of the determination of the median particle size and particle number concentration of Ag nanoparticles (AgNPs) in chicken meat. Ten laboratories from the European Union, the USA, and Canada determined particle size and particle number concentration of two chicken meat homogenates spiked with polyvinylpyrrolidone (PVP)-stabilized AgNPs. For the determination of the median particle diameter, repeatability standard deviations of 2 and 5% were determined, and reproducibility standard deviations were 15 and 25%, respectively. The equivalent median diameter itself was approximately 60% larger than the diameter of the particles in the spiking solution. Determination of the particle number concentration was significantly less precise, with repeatability standard deviations of 7 and 18% and reproducibility standard deviations of 70 and 90%.

**Electronic supplementary material:**

The online version of this article (doi:10.1007/s00216-017-0427-2) contains supplementary material, which is available to authorized users.

## Introduction

Particles with dimensions in the nanoscale (defined as 1–100 nm by ISO/TS 80004-1 [1]) may show different properties from the same chemical material in its bulk form, either due to the increased surface-to-volume ratio or the emergence of quantum phenomena at these small ranges. Such particles not only have a significant potential for improved material properties but may also have adverse effects. Legislation requiring the labeling of the presence of materials in their nanoform in cosmetics [2] and food [3] exists at the EU level. To avoid a multitude of conflicting definitions of *nanomaterial*, the European Commission has published a recommendation for a definition [4] which it intends to use in future regulation. This definition defines a nanomaterial as “natural, incidental or manufactured material containing particles, in an unbound state or as an aggregate or as an agglomerate and where, for 50 % or more of the particles in the number size distribution, one or more external dimensions is in the size range 1 nm-100 nm.”

As with many pieces of legislation, further assessing whether the requirements for labeling are implemented correctly requires effective control, based on reliable measurements. Unfortunately, of the most commonly used techniques for the determination of the diameters of nanoparticles, only electron microscopy (EM), particle tracking analysis (PTA), and single-particle inductively coupled plasma mass spectrometry (sp-ICP-MS) deliver number-based particle size distributions.

Silver nanoparticles (AgNPs) show a bactericidal effect which is exploited in, for example, making sports cloth more resistant to smell caused by bacteria. Using the same properties, shrink films, cutting boards, and storage boxes containing AgNPs are offered on the Internet [5–7]. It is conceivable that AgNPs migrate from these materials into foodstuffs. The detection of AgNPs in foodstuffs may, therefore, be important.

The concept of utilizing ICP-MS for single particle analysis and colloid suspensions was first published by McCarthy and Degueldre [8] and tested for a series of particles in aqueous suspensions, including TiO_2_ and Al_2_O_3_ [9] and Au [10].

More recently, sp-ICP-MS has been described as a tool for the determination of NPs in various applications like Au in bio-analytical samples [11, 12], Pb in airborne particles [13], or Ag in aqueous matrices [14]. The use of this technique has been reviewed by several authors [15–17]. However, as can be expected of developing techniques, data on thorough validation is sparse: Abad-Alvaro and colleagues compared the evaluation of the particle number concentration using millisecond and microsecond dwell times and achieved repeatability standard deviations of 5% (millisecond dwell times) and 1% (microsecond dwell times), respectively [18]. Witzler and co-workers performed an in-house validation study of the determination of particle size and number concentration of Ag and Au particles in water, apple juice, and orange juice [19] and achieved repeatability standard deviation for particle sizes of AgNPs of better than 8% and intermediate precisions of better than 25%. sp-ICP-MS was also among the methods investigated in the EU-funded project “NanoLyse,” which aimed at developing and validating methods for the detection and quantification of nanoparticles in food. Within this project, an interlaboratory comparison for the determination of median particle size and particle number concentration of AgNPs in food simulants (water; water/ethanol) was organized [20]. This interlaboratory comparison concluded that sp-ICP-MS was already performing satisfactorily for screening purposes when limited to particle diameter. However, this study was limited to very simple food simulants, and it was not clear whether the results would also apply to more complex materials. Another intercomparison on spherical and monodisperse gold nanoparticle in aqueous suspension was performed by Montero and colleagues [21]. They reported good agreement between the sp-ICP-MS results and other methods, with better results achieved for the 60-nm particles than for the 30-nm particles.

This manuscript describes the follow-up study of the intercomparison in food simulants: a significantly more complex matrix (chicken meat) was chosen to check the performance of sp-ICP-MS in settings that require significant sample preparation.

## Experimental

### Study concept

The present study was planned as an interlaboratory method performance study, not as a proficiency test of individual laboratories. The goal was thus to evaluate the performance of the combined sample preparation and sp-ICP-MS detection method under interlaboratory conditions. Precision was determined following the agreement of the results from the participants for the diameter determination, the particle number concentration, and the mass concentration and involved both the sample preparation and the measurement by sp-ICP-MS. As no certified reference material for nanoparticles in complex matrices is currently available, only rough estimates of the trueness could be made.

All the participants used the same standard operating procedures (SOPs) but were free in their choice of calibration standards. Deviations from the SOP had to be reported on the result sheets. The SOPs for measurement of nanoparticle suspensions by sp-ICP-MS and sample preparation of meat by enzymatic digestion were distributed to the participants, together with the instructions for the study, as well as a specific macro (in Microsoft Excel, as described in [32]) for the evaluation of the raw ICP-MS data and the calculation of the results. The participants had the opportunity to participate in either a hands-on training or remote workshop for the previous sp-ICP-MS interlaboratory study on AgNP in food simulants. Lean chicken muscle meat, spiked with AgNP, was selected as a test matrix to mimic both the migration of AgNP from food contact materials and samples obtained from toxicological studies on the oral uptake of AgNP. The diameter of the spiked AgNP of 35 nm was selected to be in a comfortable range of the sp-ICP-MS method, thus avoiding the risk that matrix interferences cause a substantial bias when working at the lower size limit of the method (ca. 20 nm according to the in-house validation of the method).

### Test samples

The test samples consisted of blank and spiked chicken muscle meat. The chicken meat homogenates were prepared from fresh organic chicken filet purchased from a local butcher and spiked with aqueous suspensions of polyvinylpyrrolidone (PVP)-stabilized Ag nanoparticles containing 0.2 and 1 g kg^−1^ Ag, respectively (Nanogap, Milladoiro, ES). This suspension consists of close-to-spherical particles with constituent particle diameters ranging from 15 to 75 nm (as determined by TEM). The median diameter as determined by sp-ICP-MS was 33 nm. In TEM, smaller particles with diameters of between 15 and 20 nm are visible, which are below the limit of detection for sp-ICP-MS [22]. The number-based equivalent median circular diameter as measured by TEM is 26–27 nm. No tests were performed on the blank chicken meat, as the Ag mass fraction in meat is several orders of magnitude below the spike levels [23]. A complete description of the preparation, homogeneity, and stability assessment of the AgNP chicken test material is given in [24]. The samples were stored at −130 °C for 6 months and sent frozen on dry ice to the participants.

The properties of the provided samples are summarized in Table 1.

**Table 1:** Properties of the chicken meat samples used in the interlaboratory study. Stated uncertainties are expanded uncertainties (coverage factor *k* = 2) corresponding to a level of confidence of about 95%

	NL13	NL14
Equivalent median diameter, TEM^a^ (nm)	27 ± 5	26 ± 2
Equivalent median diameter sp-ICP-MS^b^ (nm)	33 ± 1	Not determined
Ag mass concentration^c^ (g kg^−1^)	0.105 ± 0.004	0.547 ± 0.007
*s*_wb_ (%)	3.9	4.5
*s*_bb_ (%)	0.8	2.3
${u}_{\mathrm{bb}}^{\ast }$ (%)	1.8	2.1

^a^Number-weighted median equivalent circular diameter as determined by TEM based on measurement of 640 (NL13) and 1758 (NL14) particles

^b^Number-weighted equivalent mass diameter as determined by sp-ICP-MS

^c^Mass fraction as determined by *k*_0_NAA

Homogeneity of the Ag mass fraction was used as a proxy of the homogeneity of the Ag nanoparticles. De-mixing of smaller and larger particles in the homogenate or differences in local agglomeration (aggregation at some places and non-agglomeration in others) could mean that the mass fraction could be homogeneous while the particle diameters are not. Such effects were regarded as unlikely and concluded that the mass fraction should allow a meaningful assessment of the particle homogeneity.

Samples were homogenized and mineralized by a nitric acid/hydrogen peroxide mixture prior to Ag quantification with ICP-MS. Ten units each of NL13 and NL14 were selected from the stock. Care was taken to cover the whole batch, and each unit was analyzed in duplicate for the total Ag mass fraction by ICP-MS. The relative standard deviation within bottle (*s*_wb_) and between bottles (*s*_bb_) and the between-bottle standard deviation potentially hidden by the method repeatability (${u}_{\mathrm{bb}}^{\ast }$) were calculated according to [25], and the results are shown in Table 1. For both materials, the method repeatability limits the detectable inhomogeneity at about 2%, with the calculated between-bottle standard deviation for NL13 below this value (0.8%) and for NL14 slightly above this value (2.3%). The samples fulfill the ISO 13528 [26] requirement for sufficient homogeneity, based on a previous intercomparison on sp-ICP-MS [20], and a between-laboratory standard deviation of 20–30% was expected.

The stability of the materials was assessed by TEM twice within 6 months. The first experiments were conducted on ten different days in the time frame of less than 20 working days. Ten samples were investigated in duplicate, and ten TEM images were recorded per sample that was taken. The later experiment included two ampoules per material measured in duplicate. The sample preparation and imaging for this test was each carried out on the same day, and three images were taken per replicate. No significant difference was observed between the measurements at the different time points. Also, the particle number values obtained by TEM were constant with about 1 and 5 particles μm^−2^ for NL13 and NL14, respectively, confirming sufficient stability under the chosen storage conditions over the period of 0.5 year. However, it must be stated that the median particle diameters in the meat samples were 6 nm smaller than in the spiking dispersion, indicating some dissolution during material processing.

In conclusion, the available homogeneity and stability data demonstrated that homogeneity and stability were sufficient not to affect the between-laboratory variation for the investigated parameters.

### Participants

The same laboratories that participated in the previous intercomparison for sp-ICP-MS of Ag nanoparticles in food simulants [20] were also invited to participate in this study. Ten of the 23 laboratories participating in the study of food simulants were also interested in participating in this study. Eight of the laboratories were from five different European countries, and one laboratory each came from the USA and Canada. Prior to the first sp-ICP-MS interlaboratory study on AgNP in food simulants, eight of them had experience with sp-ICP-MS, albeit in some cases only for a short time, or were in the process of establishing the methodology in their laboratories. Two laboratories had prior experience in the measurement of NPs with conventional ICP-MS. All ten laboratories had participated in the first sp-ICP-MS interlaboratory study on AgNP in food simulants. Nine participants submitted valid results.

### Measurement procedure

Detailed SOPs for sample preparation and sp-ICP-MS analysis were sent to all the participants. Details of the method were published by Peters et al. [27]. In brief, the meat homogenate was incubated with protease K at 37 °C for 3 h and then diluted according to the requirements of the sp-ICP-MS method. The detection method consists of setting the ICP-MS dwell time to 3 ms with a total acquisition time of 60 s, giving 20,000 separate bins. Transport efficiency is determined using the frequency method [28] and using Au nanoparticles (NIST RM 8013). If the samples are highly diluted, the presence of two particles in the plasma at the same time is unlikely. Using data from a Poisson distribution reveals that, if on average 2000 particles are detected over this 20,000 bins, the fraction of events arising from doublets/triplets is below 5%. A simulation using MS Excel shows that these multiplets introduce a bias of <0.15 nm for 20- and 40-nm particles which is negligible. Details on the instrument settings used by the participants are given in the Electronic Supplementary Material (ESM).

Ionic Ag standards in aqueous solution with concentrations between 0.2 and 5 μg L^−1^ were prepared. Calibration with these standards allows for the calculation of the mass of each particle. The nebulization efficiency, a prerequisite for the correct mass and particle number quantification, is determined using Au nanoparticles of known concentrations using the frequency method as described by Pace and co-workers [28]. The samples are diluted to fall within the calibration range of the method. The analytical method was validated at RIKILT according to 2002/657/EC, and performance characteristics are in accordance with this Commission Decision. The threshold between background and particles was determined as the minimum in a frequency distribution of the measured signal intensities. For Au and Ag particles, this minimum is very clear and small deviations to lower and higher signal intensities only have a minimal effect on particle size. At the time of the execution of the study, no commercial software for the evaluation of sp-ICP-MS was available. Therefore, a dedicated Excel spreadsheet was developed by RIKILT and provided to all participants. This is the same spreadsheet used in the earlier interlaboratory comparison of AgNPs in food simulants, thereby acquainting the participating laboratories with it [20].

### Study protocol

Each participant received six sample vials, two blank chicken meat homogenates, two vials of homogenates spiked at level 1 (NL13; approx. 0.1 g kg^−1^), and two vials of chicken homogenate spiked at level 2 (NL14; approx. 0.5 g kg^−1^). The samples were stored in liquid nitrogen (below −150 °C) since processing (6 months before this intercomparison). Also, one vial of protease K (25 mg) was provided. The chicken samples had to be stored frozen until their analysis. Chicken homogenates should be thawed only immediately before use. After thawing, the contents of each vial had to be well mixed with a spatula.

The following measurements were performed:

- Measurement of two blank chicken meat samples, one at the start and one at the end of the analytical run
- Preparation and measurement of spiked control samples using the blank chicken homogenate and a 60-nm monodisperse Ag nanoparticle suspension of their choice as described in the “Particle number concentration” section of the sample preparation SOP; each spiked control sample had to be measured once. Information on the source of the AgNPs for each laboratory is given in the ESM
- Three measurements each on NL13 and NL14; each of the three measurements came from a different sample preparation/different subsample. The three subsamples per material were taken from the same vial (the second vial served as spare in case of accidents)

The participants were asked to report the results for median particle diameter, Ag particle mass concentration and particle number concentration, as well as the instrumentation and any deviations from the SOPs on the provided spreadsheet that was returned both electronically, as well as a signed hardcopy.

### Data evaluation

Evaluation of the remaining results was performed according to ISO 5725-2 “Accuracy (trueness and precision) of measurement method and results” [29]. Results were screened using the graphical consistency technique (Mandel’s *h* and *k*-plots) to test for consistent bias or high variances, and laboratories with consistently high bias or variances were excluded. The remaining datasets were tested for outlying means and variances using the Grubbs and Cochran procedure. Outliers at a 99% significance level were removed from the dataset. The remaining datasets were tested for normality using normal probability plots and skewness and kurtosis tests. Absolute and relative repeatability standard deviation (*s*_r_, *rs*_r_), absolute and relative standard deviation between laboratories (*s*_L_, *rs*_L_), and absolute and relative reproducibility standard deviation (*s*_R_, *rs*_R_) were calculated from the mean squares within group (MSW) and means squares between groups (MSB) using one-way analysis of variance (ANOVA)

1 $$ {s}_{\mathrm{r}}=\sqrt{\mathrm{MSW}}\kern2em {rs}_{\mathrm{r}}=\frac{s_{\mathrm{r}}}{\overline{\overline{x}}} $$

2 $$ {s}_{\mathrm{L}}=\sqrt{\frac{\mathrm{MSB}-\mathrm{MSW}}{3}}\kern2em {rs}_{\mathrm{L}}=\frac{s_{\mathrm{r}}}{\overline{\overline{x}}} $$

3 $$ {s}_{\mathrm{R}}=\sqrt{s_{\mathrm{r}}^2+{s}_{\mathrm{L}}^2}\kern2em {rs}_{\mathrm{R}}=\frac{s_{\mathrm{r}}}{\overline{\overline{x}}} $$

where $\overline{\overline{x}}$ is the grand mean of all results.

The data evaluation was performed using the characterization module of the program SoftCRM [30].

## Results and discussion

Nine of the ten participants submitted valid results. One laboratory could not detect particles: the modal particle diameter decreased with dilution from 55 nm at a dilution of 1:3000 to 30 nm at a dilution of 1:10,000. The laboratory, therefore, suspected that the silver nanoparticles were destroyed by the preparation procedure to give an ion-like behavior in the analyzed dispersion.

Laboratory 14 obtained an equivalent median diameter of 90 nm for the blank material spiked with 60-nm nanoparticles. Also, the diameter standard deviation obtained for this rather monodisperse material was much higher than the other participants. The laboratory was, therefore, excluded from the further evaluation.

The results are presented individually for the determined parameters mean particle diameter, AgNP number concentration, and Ag particle mass concentration in the following sections.

### Selectivity

Three laboratories reported particles in the blank chicken homogenate. The Ag particle mass fractions reported for the blanks were for two of them, a factor 1000–2000 below the one reported for NL13, but the particle mass fraction reported by the third was only a factor 21 below the ones found in NL13. This highlights that sp-ICP-MS can certainly distinguish between AgNPs and remaining matrix, but false positive can occur, which could be caused by carryover [31].

### Median particle diameter

**Fig. 1:**
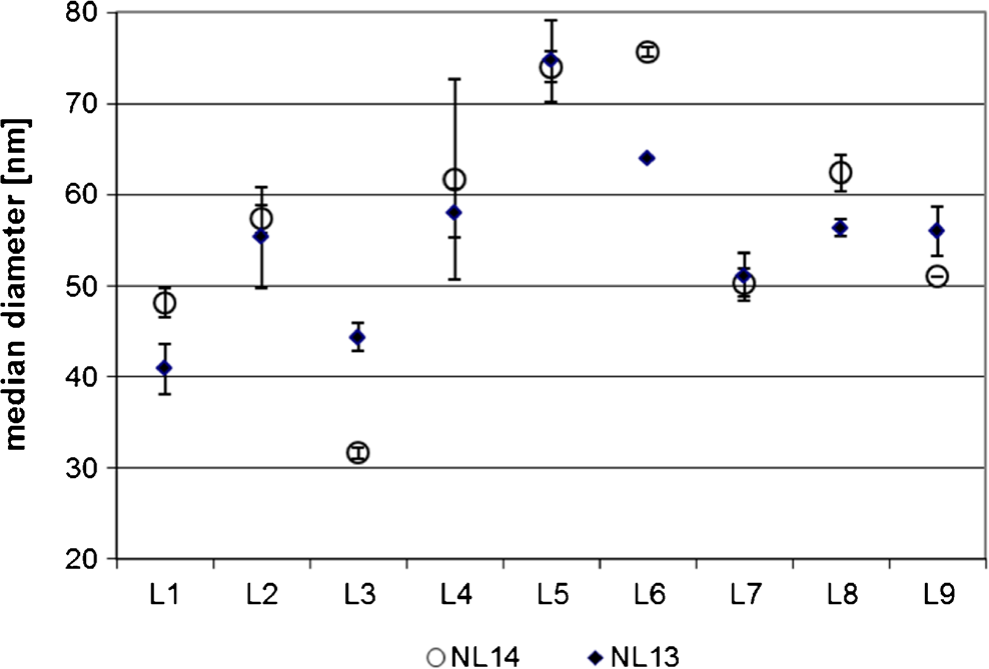
Data obtained for the equivalent median diameter. Shown are the laboratory means and standard deviations (*n* = 3) for each laboratory. *Full diamonds* NL13; *open circles* NL14

Figure 1 displays the results obtained for the measured median particle diameter. The data obtained for the two materials are very similar. This is obvious because both materials were produced from the same blank chicken paste spiked with the nominally equivalent AgNP suspension.

Mandel’s *k*-plot for within-laboratory variation did not indicate that any laboratory had consistently large standard deviations, but the result of L4 for NL14 was flagged as an outlier of both using the critical value of the Mandel *s* statistic and the Cochran procedure at a 99% confidence level. This result was, therefore, removed. None of the results consistently showed significant deviations from the grand mean.

**Fig. 2:**
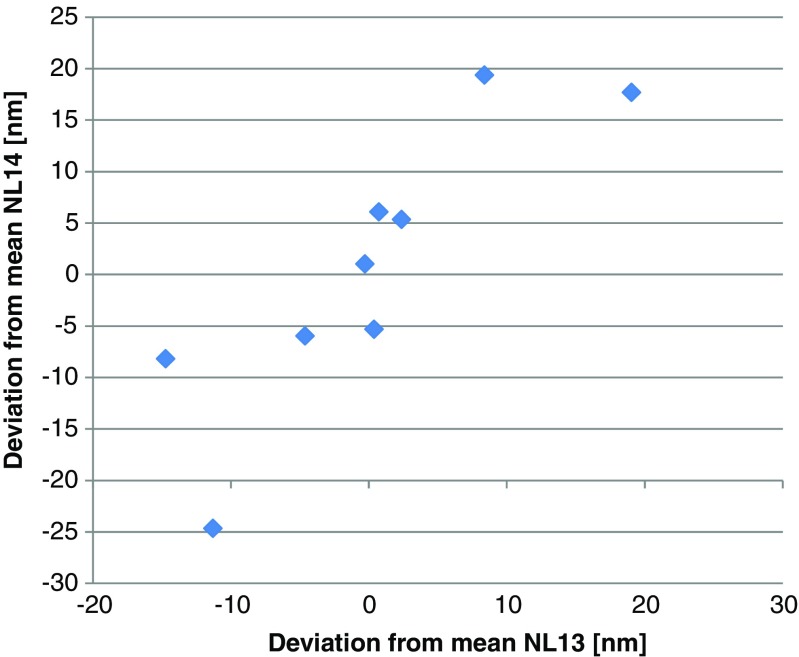
Youden plot for the median particle diameter. The diamonds show for each sample the  deviation of the average of each laboratory from the grand mean

The Youden plot (Fig. 2) clearly shows that most deviations are systematic in nature, with four laboratories each showing a positive and negative deviation.

The data, after removal of L14 and the result for NL14 of L4, followed a Gaussian distribution. Repeatability of the data was very good (better than 6%) and is comparable with what has been reported for the original development of the method [27]. The between-laboratory standard deviations were with 14 and 25%, significantly larger and are also a factor 3 to 5, larger than the within-laboratory repeatability reported [27]. All statistical data are listed in Table 2.

**Table 2:** Statistical data for the equivalent median diameter, particle number concentration, and Ag mass fraction

	Equivalent median diameter			Particle number concentration			Ag mass fraction		
	Spike 60 nm; 0.1 g kg^−1^	NL13	NL14	Spike 0.1 g kg^−1^	NL13	NL14	Spike 0.1 g kg^−1^	NL13	NL14
*N*	7	8	8	7	8	8	7	8	8
Outliers	0	0	1	0	3	0	0	2	0
Mean	59.3 nm	53.2 nm	53.8 nm	24.9 · 10^9^ kg^−1^	9.6 · 10^9^ kg^−1^	56.2 · 10^9^ kg^−1^	0.044 g kg^−1^	0.022 g kg^−1^	0.181 g kg^−1^
*s*_r_ (*ν* = 16)	Not calculated	2.8 nm	1.3 nm	Not calculated	0.65 · 10^9^ kg^−1^	10.3 · 10^9^ kg^−1^	Not calculated	0.0040 g kg^−1^	0.049 g kg^−1^
*r*s_r_ (*ν* = 16)	Not calculated	5.2%	2.4%	Not calculated	6.8%	18%	Not calculated	18%	27%
*s*_L_ (*ν* = 7)	Not calculated	7.4 nm	13.6 nm	Not calculated	6.7 · 10^9^ kg^−1^	48.4 · 10^9^ kg^−1^	Not calculated	0.0070 g kg^−1^	0.108 g kg^−1^
*rs*_L_ (*ν* = 7)	Not calculated	13.9%	25.3%	Not calculated	70%	86%	Not calculated	32%	60%
*s*_R_	3.6 nm	7.9 nm	13.6 nm	28.8 · 10^9^ kg^−1^	6.7 · 10^9^ kg^−1^	49.5 · 10^9^ kg^−1^	0.042 g kg^−1^	0.008 g kg^−1^	0.119 g kg^−1^
*rs*_R_ (%);	6.1	14.8	25.3	116	70	88	95	36	66

*N* = Total number of technically accepted results; *ν* = degrees of freedom; no *s*_r_, *rs*_r_, *s*_L_, and *rs*_L_ could be calculated for the spike as only one result per laboratory was reported. The equivalent median diameter as determined by TEM of the AgNPs in the suspension used for the preparation of NL13 and NL14 was 30 ± 6 nm (NL13) and 32 ± 3 nm (NL14) (expanded uncertainties; *k* = 2 [24]). Estimated approximate particle number concentrations as determined by TEM are 1 · 10^10^ g^−1^ (NL13) and 1 · 10^10^ particles g^−1^ (NL14)

The average median particle diameter itself is with 53.2 and 53.8 nm significantly larger than the diameters reported by Loeschner and co-workers for the spiked suspension and freshly spiked chicken meat when using TEM and sp-ICP-MS (30–35 nm) [22] or AF4-ICP-MS (37 nm; [31]).

One explanation for the different size could be the ionic calibration for Ag, as an underestimation of ICP-MS response/sensitivity will lead to an overestimation of the particle diameter. However, the ionic calibration was performed in solutions in ultrapure water and the samples themselves were diluted in ultrapure water. As a 50% underestimation of the sensitivity only results in an overestimation of the particle diameter by 25%, it is unlikely that matrix effects alone can explain this difference.

A too high size limit of detection of the sp-ICP-MS method will result in a bias towards larger equivalent median diameters. Indeed, previous work [32] as well as the interlaboratory study for sp-ICP-MS in food simulants [20] indicated a size limit of detection (LOD) of about 20 nm. Eliminating all particles below 20 nm from the TEM measurements only increases the median particle diameter by 1 nm. The size LOD of the sp-ICP-MS would have to be 45 nm (thus not detecting 90% of particles from the TEM dataset) to reconcile the sp-ICP-MS data with the TEM data, which is unlikely.

The most likely reason for the differences observed could be the sample preparation protocols resulting in different degrees of de-agglomeration: measurements by TEM [33] show a tailing distribution, indicating the presence of small agglomerates, which is also confirmed by field-flow fractionation (FFF)-ICP-MS [34]. The sample preparation by TEM (dilution + probe sonication + ultracentrifugation) may cause some degree of de-agglomeration. The procedure used in this work relied on a long (3 h at 37 °C) digestion with a relatively low enzyme mass fraction (4 μg mg^−1^ tissue) whereas Loeschner et al. [31] used a much shorter (40 min at 37 °C) digestion using a much higher enzyme concentration (60 μg mg^−1^ tissue). This higher enzyme concentration results in de-agglomeration of particles as shown by AF4-ICP-MS, where the stable doublets and triplets of the AgNP suspension [34] disappear after spiking to meat and enzymatic digestion [22]. The combination of lower enzyme concentration/longer digestion time may therefore have preserved the original agglomeration state and/or might have led to additional agglomeration. This means that each sample preparation protocol potentially changes the agglomeration states, and it is impossible to decide on a *true* nominal value for the material in question.

The result of the chicken material freshly spiked with 60-nm AgNPs used as quality control also confirmed the general suitability of the method: seven laboratories submitted data, six of which reported median diameters between 57 and 69 nm and one reported a value of 90 nm.

Interestingly enough, no systematic deviation for the spike was detected. The average of all the results agreed with the nominal value of 60 nm. The standard deviation between laboratories was with 6%, also significantly smaller than that for NL13 and NL14. This may be due to the monodisperse nature of the spike but could also be due to the different surface functionalization (citrate stabilized instead of PVP stabilized) and/or the larger size. This good agreement between the nominal value of the equivalent median diameter of the spike was combined with a significant broadening of the measured particle size distributions: laboratories reported standard deviations of the particle size distributions between 1.5 and 55 nm (2.5–92%), whereas the specification of one of the producers was <9% (nanoComposix; no specification for the AgNPs from Sigma). These data show that significant broadening of the distribution occurred.

### Particle number concentration

**Fig. 3:**
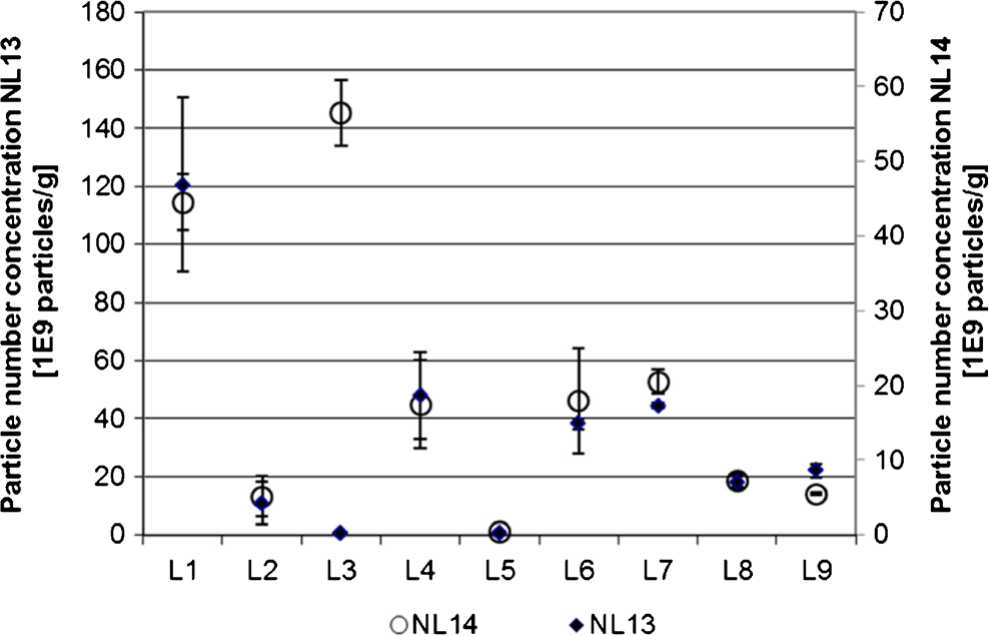
Data obtained for the particle number concentration. Shown are the laboratory means and standard deviations (*n* = 3) for each laboratory. *Full diamonds* NL13; *open circles* NL14

The results reported for the particle number concentrations are shown in Fig. 3. Results for the particle number concentration varied over 2 (NL14) to 3 (NL13) orders of magnitude. As can be seen in Fig. 3, the visual pattern is identical for both materials, showing a strong laboratory-dependent effect.

For NL13, L1 was flagged as an outlying average and L1, L10, and L12 were flagged as Cochran outliers. No outlying means or averages were detected for NL14. After removal of the outliers, the data followed a Gaussian distribution.

Relative repeatability standard deviations of the particle number concentrations were with 7% (NL13) and 18% (NL14) acceptable (see also Table 2). The between-laboratory standard deviations ranged from 70 to 90% and showed a severe lack of reproducibility of the method.

Despite the apparently laboratory-specific nature of the bias, normalization to the particle number concentration obtained for the spiked samples did not improve the precision. Between-laboratory standard deviations even increased to above 100%.

A number of 32 and 83 particles per TEM image were reported by Dudkiewicz and colleagues for NL13 and NL14, respectively, who also estimated that each replicate corresponded to 2.8 · 10^−9^ mL [33]. Using a bulk density of chicken breasts of 1.1 g mL^−1^ [35], this would correspond to particle number concentrations of 1 · 10^10^ g^−1^ (NL13) and 3 · 10^10^ particles g^−1^ (NL14). Interestingly enough, several participants reported significantly higher particle number concentrations.

A second attempt of assessing trueness was made using the data obtained for the spiked blank homogenates: participants were instructed to spike the homogenates with 0.1, 0.2, and 0.5 g kg^−1^ Ag nanoparticles of a nominal diameter of 60 nm. This corresponded to 84 · 10^9^, 170 · 10^9^, and 421 · 10^9^ particles g^−1^. Compared with these target values, the laboratories recovered, on average, 19% of the particles present in the samples. A similarly low particle number recovery for this material (20%) had been reported by Loeschner et al. [31], who suggested a mixture of dissolution and chemical transformation which, while apparently not changing the particle size distribution significantly, seems to make many particles not accessible to sp-ICP-MS anymore. Peters et al. [27] reported recoveries of ca. 40% of 60 nm AgNPs spiked to chicken meat after storage for 48 at 4 °C in the dark. While the particle diameter decreased only slightly from 60 to 56 nm, SEM/EDX analysis of the digest showed a much smaller AgNP particle number, and the presence of silver sulfide, confirming chemical translation of AgNP particles. This may also have happened with the spiked materials in this study.

Uncertainties in the transport efficiency may contribute to the bias: the calculated particle number concentration is the product of the measured particle number and the transport efficiency. The latter depends on the particle number concentration of the reference suspension used for the determination of the transport efficiency. Here, a 50% lower particle number concentration in the reference suspension will lead to a 50% lower particle number concentration measured in the chicken meat. However, such a deviation cannot explain a recovery of only 20% of the AgNPs in these materials.

By considering the poor precision and the much bias, it is concluded that the method is not yet suitable for a reliable determination of particle number concentrations.

### Particle mass concentration

**Fig. 4:**
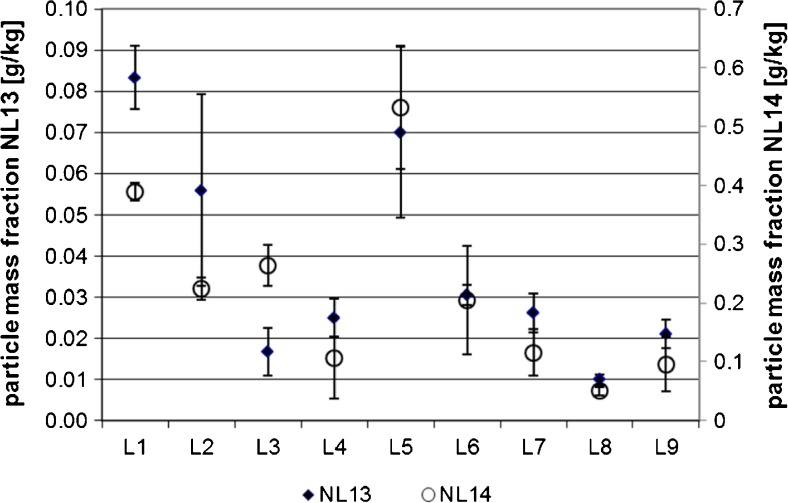
Data obtained for the particle mass fraction. Shown are the laboratory means and standard deviations (*n* = 3) for each laboratory. *Full diamonds* NL13; *open circles* NL14

The particle mass concentration is calculated by summing up the mass of all individual particles detected in sp-ICP-MS, i.e., excluding ionic Ag. As the particle mass fraction is calculated from the particle number concentration, the distribution of results for the particle mass fraction for NL13 and NL14 also follows the same pattern for all laboratories (see Fig. 4).

For NL13, L10 was flagged as an outlier of variance and L1 was flagged as an outlier of means. These data were removed from the analysis. No outliers of variance were found for NL14. After removal of outliers, all the data follow Gaussian distributions and the statistical parameters are listed in Table 2.

Repeatability standard deviations of the Ag mass fraction are significantly worse than for the particle number concentration, but the between-laboratory standard deviations are lower, resulting in slightly lower reproducibility standard deviations.

The mean of the Ag particle mass fractions is only 21% (NL13) and 33% (NL14) of the known spike mass fraction. This low finding is presumably partly due to dissolution during sample storage and sample preparation, as some laboratories reported an ionic higher Ag background and could also be caused by poor overall recovery. One of the laboratories reported a mass recovery of the spike of 39%, which is roughly in line with the recovery for NL13 and NL14.

Given the high dilutions used by most participants, it is unlikely that differences in the sensitivity between the Ag standard and the diluted digests are the reason for the low mass recoveries. Besides dilution, agglomeration/aggregation is expected to result in lower detected Ag mass fractions. Large agglomerates/aggregates (few 100 nm size) contain a large mass but their number is very low. The likelihood of detecting such a large *particle* during one measurement is low. Further, large agglomerates/aggregates are likely to settle during sample preparation/analysis.

## Conclusions

The interlaboratory study showed promise for the determination of the median particle diameter: reproducibility standard deviations between 15 and 25% are not unusual and are deemed acceptable for the determination of many contaminants in food. Bearing in mind that the AgNP mass fraction in the samples in this study was rather high (0.1 and 0.5 g kg^−1^), further studies are needed to evaluate whether this level of agreement can also be obtained at the lower AgNP mass fractions, more likely to be caused by contamination from food contact materials.

Determination of AgNP mass fractions and AgNP number concentrations is currently neither reproducible nor true enough for the application to complex real-world samples. Further method improvement is needed to eliminate bias and to arrive at a more robust method.

It should be noted, however, that some of the observed reproducibility issues may be associated with the chemical reactivity of AgNP. Silver easily transforms by oxidation (dissolution of particles), complexation, association to proteins, de novo particle formation from dissolved Ag (AgCl, Ag_2_S, Ag^0^) under the experimental conditions and are not inherent to sp-ICP-MS as a technique.

The difference between the sizes obtained in this work and those obtained using a different enzymatic digestion protocol [31] showed that sample preparation might have a large influence on the result obtained. In particular, the possibility of agglomeration and de-agglomeration during sample preparation should be borne in mind. The difference between the behavior of the (in most cases) citrate-stabilized AgNP spike in the freshly spiked chicken samples (the same size as spiking solution) and the aged samples spiked with PVP-stabilized AgNPs (60% larger diameter than that in spiking solution) demonstrated that the obtained results might differ depending on the age of the sample, as well as on the stabilizing agent for the AgNPs, with the age of the samples being the more likely cause for the observed differences. This means that for unknown samples, additional tests may be needed to confirm that the sample preparation is adequate for the AgNP/matrix combination in question and the age of the samples. Particle stabilization (e.g., with surfactants) during or after enzymatic digestions should be considered in future work to avoid the formation of large aggregates after degradation of the matrix. It might also be necessary to dilute with solutions containing stabilizers instead of ultrapure water. In method validation (as proposed in [36]), ideally, a broad spectrum of differently coated NPs should be investigated. It might not be possible to define a single sample preparation protocol applicable for all nanoparticle types and capping agents. In this case, simultaneous measurement of samples containing different nanoparticles or nanoparticles with unknown coatings would be difficult to impossible.

## Electronic supplementary material

ESM 1 (PDF 47.9 kb)
