# Supplementary material for: Results of an interlaboratory method performance study for the size determination and quantification of silver nanoparticles in chicken meat by single-particle inductively coupled plasma mass spectrometry (sp-ICP-MS)
Source: Anal Bioanal Chem. 2017 Jun 20;409(20):4839–48. doi: 10.1007/s00216-017-0427-2 (PMC5519662; doi:10.1007/s00216-017-0427-2)
Supplement: Supplementary file 1 — (PDF 47.9 kb) [file 216_2017_427_MOESM1_ESM.pdf]

Analytical and Bioanalytical Chemistry

Electronic supplementary material

Results of an interlaboratory method performance study for the size determination and quantification of silver nanoparticles in chicken meat by single-particle inductively coupled plasma mass spectrometry (sp-ICP-MS)

Stefan Weigel, Ruud Peters, Katrin Loeschner, Ringo Grombe, Thomas P.J. Linsinger

Table S1 AgNPs used by the individual laboratories for spiking and recovery

Laboratory	Nanoparticle
L1	Sigma Aldrich-730815; no Lot # given
L2	Sigma-Aldrich 730815;Lot: MKBL3477V
L3	Sigma Aldrich 730815; Lot MKPP8300V
L4	NanoComposix 60 nm Citrate BioPure Silver (Lot Number DAG1224)
L5	NanoComposix, 60 nm nanospheres, biopure 1 mg/l, citrate. Lot# DAG1224.
L6	Nanocomposix 60nm citrate Biopure Silver Lot. DAG1720
L7	Nanocomposix EAW1093
L8	NanoComposix, Lot#: DAG1224
L9	Ted Pella 60nm Pelco Citrate NanoXact Silver Lot number DAC1183

Table S2 Method information for the individual laboratories

	L1	L2	L3
Plasma RF power forward	1200 W	1100 W	1310 W
Plasma RF power reflected	-		0W
Plasma gas flow	1.5 L/min	15 L/min	13 L/min
Auxillary gas flow	1.2 L/min		0.7 L/min
Nebulizer gas flow	1.0 L/min	0,89 L/min	0.92 L/min
Nebulizer	Glass concentric Slurry	Meinhard	concentric nebulizer
Spray chamber	Baffled cyclonic, glass	Cyclonic spray chamber, glass	conical spray chamber, quartz
Sampling cone	Pt	Ni	Ni
MS resolution	0.7 amu	Not given	Not given
MS dwell dime	5 ms	3 ms	3
Comments	The stock nanoparticle standard used for spiking and recovery was 20 mg/l. A spiked control of 0.1 g/kg was prepared by adding 1 ml of the stock standard instead of 20 ul	1. Use of 100nm instead of 60nm Ag-Nanoparticle--> conc(Ag) = 20 mg/l --> (Spike-conc.: 0,02 / 0,05 / 0,1 g/kg)<br>--> Recovery (Particle size): 92±6 nm --> Recovery (mass conc.): 39%<br>2. Sample preparation in an ultrasonic bath (ice-cooled water)<br>3. Incubation at 37°C in an oven while permanently shaking	Little deviation of ICP-MS parameters is showed as above. Silver Nanoparticles with nominal diameter of 60 nm from Sigma (0.02 mg/mL) were used for the positive control. Thus the injection volume of silver nanoparticles in the positive control was adjusted accordingly to get the mass ratio required.
Dilution NL13	83818	2 x 10000, 1 x 20000	1000
Dilution NL14	246066	20000	100000

	L4	L5	L6
Plasma RF power forward	1400	1600	1300 W
Plasma RF power reflected	Value not available for NEXION 300 D	---	not available, self adjustable free running generator
Plasma gas flow	13 L/min	18 L/min	15.5 L/min
Auxillary gas flow	0.7 L/min	1.2 L/min	1.20 L/min
Nebulizer gas flow	0.99 L/min	0.99 L/min	1.00 L/min
Nebulizer	Meinhard Type A 0.5	PFA, low flow	MicroFlowPFA nebulizer
Spray chamber	Cyclonic, quartz	Cyclonic, quartz	Apex IR (Elemental Scientific, Omaha, NE)
Sampling cone	Ni	Pt	Pt
MS resolution	0.7 amu	<1 amu	0.727 amu
MS dwell dime	3 ms	3 ms	3 ms
Comments	Screw cap 15 mL PE test tube were used for sample preparation. In both worksheet "Calibration" and "Sample" the cell values in the "signal (cps)" column that exceeded the total acquisition time were replaced with 0. Difficulties were found on selecting the "limit for particle detection" point due to high background (dissolved Ag or continuos NPs population?). In order to investigate the reasons of that we performed TEM analysis on sample NL 14-2 (median 25.8 nm; MAD 4.3 nm). Size standard deviation not evaluated (single measurement performed).	used ESI one fast sample introduction without internal standard line. NL13 measurement limited by high background ionic Ag at 2500 x dilution, used 5000 x dilution with 120 particlces detected.	Samples have been sonicated at 30 W for 5 minutes since this was the minimum applicable according to the instrumental power control settings. Indirect sonication was tentatively tried with unsatisfactory results in term of median particle size.
Dilution NL13	20	5000	100000
Dilution NL14	20	25000	750000

	L7	L8	L9
Plasma RF power forward	1404 W	1400 W	1550 W
Plasma RF power reflected	4W		11 W
Plasma gas flow	13 L/min	13 L/min	1.01 L/min
Auxillary gas flow	0.7 L/min	0.7 L/min	0.10L/min
Nebulizer gas flow	1.0 L/min	1.1 L/min	Not given
Nebulizer	Burgener Mira Mist Nebulizer (PEEK)	Concentric, glass	Micro Mist quartz
Spray chamber	impact bead; quartz	Cyclonic, glass	Schott, quartz
Sampling cone	Pt	Ni	Pt
MS resolution	Standard	0.7 amu	0.6 amu
MS dwell dime	3	3 ms	3 ms
Comments	Of the spiked control samples only the 0.1 g/kg sample was prepared and analysed	Following the digestion procedure, there were a few pieces of meat remaining undigested by the proteinase K (even after increasing the incubation time). So, immediately after vigorously vortexing the digest, an aliquot of the suspension was pipetted and diluted for spICPMS measurements. Consequently, if the remaining fragments contain Ag (which is likely the case), then we conclude that our calculated mass concentrations will be biased.	1) sonication step was performed using a vial tweeter at 0.1 cycle - 50% Amplitude in ice for 5 minutes<br>2) the AgNPs used for spiking into chicken meat were at concentration of 20 mg/L Therefore the spike is at lower concentration than 0.1g/Kg and exactly 0.02 g/kg;3) we performed for each sample and blank one digestion and from the digestion stock prepared 3 independent dilutions (triplicates)
Dilution NL13	100000	10000	55760
Dilution NL14	500000	40000	274045
